# The Fra-1: Novel role in regulating extensive immune cell states and affecting inflammatory diseases

**DOI:** 10.3389/fimmu.2022.954744

**Published:** 2022-08-11

**Authors:** Yu-Yao He, Hai-Feng Zhou, Lu Chen, Yan-Ting Wang, Wan-Li Xie, Zhen-Zhen Xu, Yue Xiong, Yi-Qi Feng, Guo-Yang Liu, Xia Li, Jie Liu, Qing-Ping Wu

**Affiliations:** Department of Anesthesiology, Union Hospital, Tongji Medical College, Huazhong University of Science and Technology, Wuhan, China

**Keywords:** FRA-1, AP-1, immune system, inflammation, tissue homeostasis

## Abstract

Fra-1(Fos-related antigen1), a member of transcription factor activator protein (AP-1), plays an important role in cell proliferation, apoptosis, differentiation, inflammation, oncogenesis and tumor metastasis. Accumulating evidence suggest that the malignancy and invasive ability of tumors can be significantly changed by directly targeting Fra-1. Besides, the effects of Fra-1 are gradually revealed in immune and inflammatory settings, such as arthritis, pneumonia, psoriasis and cardiovascular disease. These regulatory mechanisms that orchestrate immune and non-immune cells underlie Fra-1 as a potential therapeutic target for a variety of human diseases. In this review, we focus on the current knowledge of Fra-1 in immune system, highlighting its unique importance in regulating tissue homeostasis. In addition, we also discuss the possible critical intervention strategy in diseases, which also outline future research and development avenues.

## 1 Introduction

In 1988, Cohen DR and Curran T screened rat c-DNA library with FOS DNA probes, and isolated a new cDNA very similar to FOS, which was named FOS Like 1(FOSL1) (1). By the same method, Matsui et al. confirmed in 1990 that FOSL1 existed in human cells, which was 90% similar to that in rats (2). Fra-1, encoded by FOSL1, is one of the members of the FOS family in activator protein(AP-1). The AP-1 complex results from dimerization between members of JUN(c-Jun, JunB, JunD), FOS(c-Fos, FosB, Fra-1, and Fra-2), ATF(Activating Transcription Factor)(ATF2, ATF3/LRF1, B-ATF), and MAF(musculoaponeurotic fibrosarcoma)(c-MAF, MAFA, MAFB, MAFG/F/K, NRL) (3, 4). A large number of studies have shown that the contribution of AP-1 to determining cell fate depends mainly on the relative abundance of AP-1 subunits, dimer composition, quality of stimulation, cell type and cell environment. As a transcription factor, AP-1 controls genes encoding key cellular regulators. Its members can be quickly assembled into dimers by phosphorylation or methylation activation, then bind to the relevant sites of the target genes, leading to the promotion or inhibition of gene expression. At the cellular level, it involves controlling proliferation, differentiation, apoptosis and responding to environmental signals; at the biological level, it plays a vital role in organogenesis, immune response and cognitive function (5–9). The diversity of AP-1 functions makes it very difficult to be studied, and hinders the answers to some basic questions about it. Therefore, a clear understanding of the mechanism of AP-1 members is of great help to further study AP-1.

The immune system plays a key role in most diseases. It is very important to find the key factors regulating immune responses for the pathogenesis and development of various diseases. Several studies have proved the importance of AP-1 signal in immune cell reactions such as macrophages. Meanwhile, the promoter of many cytokines contains AP-1 binding sites, but the specific mechanism is still in the exploratory stage, especially Fra-1. Studies on Fra-1 are mostly focused on the proliferation, apoptosis, differentiation and transformation of cancer cells. The mechanism of its function in bone and tumor biology has been basically established (10, 11), while the properties of immune cell proliferation, activation and differentiation are rarely studied. With the development of technology, the inherent idea that Fra-1 mostly binds to the promoter of target gene to regulate the expression of the target gene has also been questioned (12, 13). Like AP-1, the function of Fra-1 is also affected by many factors. Existing studies have described Fra-1 as a positive regulator of gene transcription involved in innate immunity (14), as well as an inhibitor of gene expression in relevant contexts (15, 16). This may relate to the differences of transcription complex which is consist of Fra-1, as well as the different environment that the cells in or the different stimulations it received, also depending on the type of cells and their initial concentration in the cells. For example, in the case of Th17 cells, Fra-1 has the opposite effect on differentiation in mouse and human cells (17, 18). In addition, in different disease models Fra-1 regulates IL-6 secretion differently even in the same cells (19–21). Interestingly, the role of Fra-1 is related to its cellular localization. Fra-1 trapped in the cytoplasm inhibits Type I interferon responses to Malaria and Viral infections (22), also might be a driving force for IL-8 overexpression (23). Therefore, a comprehensive identification of the role of Fra-1 in immune processes may help to delineate new ways to ameliorate immune-related diseases such as infections, cancer, and cardiovascular diseases.

The earliest and most studied Fos family protein is c-Fos, part of the reason may be that c-Fos knockout does not cause mice to die, while fetuses lacking Fra-1 suffered severe growth retardation due to obstructed placental angiogenesis, resulting in mouse death between E10.0 and E10.5 (24). The importance of Fra-1 in embryonic development made it difficult to study, but the subsequent emergence of specific knockout and “omics” technology made the function of Fra-1 gradually clear. Here, we discuss the structural characteristics and expression regulation mechanism of FOSL1. In addition, we reviewed and summarized some studies on the role of Fra-1 in innate and adaptive immune regulation, as well as its influence on the occurrence and development of some related diseases.

## 2 The identity of Fra – 1: Structure and expression

### 2.1 Fra-1 structure

**Figure 1:**
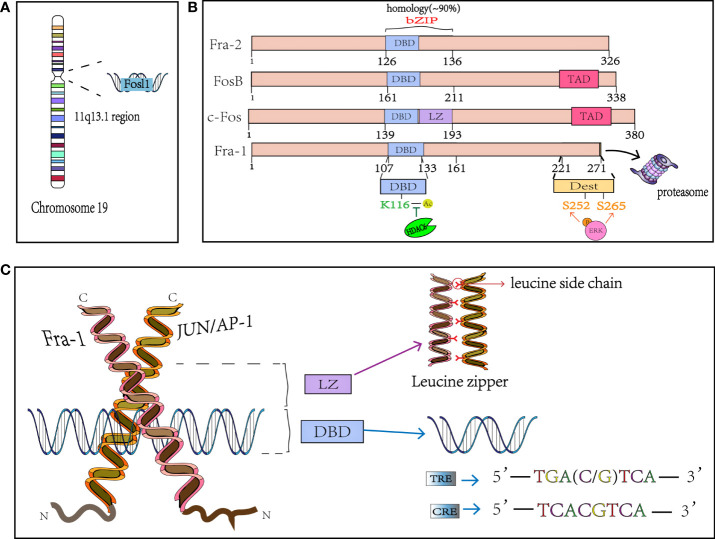
The structure of Fra-1. **(A)** FOSL1 is on chromosome 19 and located in the 11q13.1. **(B)** Schematical presentation of the structure of FOS mambers. bZIP, basic Leucine zipper region for dimerisation and DNA-binding and the FOS family has 90% homology in this region; The phosphation of Fra-1 at side S252 and S265 inhibits the COOH-terminal destabilizer increasing Fra-1 half-time and the K116 deacetylation positively controls DNA binding and transactivation. DBD, DNA binding domain; LZ, leucine zipper; TAD, C-terminal transactivating domain. DEST, destabilizer element; HDAC6, Histone deacetylase 6. **(C)** X-ray structure of the bZIP dimmer AP-1 bound to DNA; Fra-1 and Jun preferentially binds to the DNA motif known as the TRE and CRE. TRE, TPA reaction element; CRE, cAMP-responsive element.

FOSL1 was initially found to be highly expressed in many cancer cells and was defined as a proto-oncogene, which was located at locus 11q13.1 and encoded a mature mRNA with a length of 1.7kb (**Figure 1A**). FOSL1 encodes a 271 amino acid protein Fra-1 with a relative molecular mass of 29000, and the genome structure consists of four exons and three introns (4, 25). Fra-1 is one of the members of FOS subfamily in the nuclear transcription factor AP-1 family (3), shares the common structural characteristics of AP-1. Like other members of AP-1, Fra-1 is also known as the basic Leucine zipper (bZIP) protein, where the leucine zipper motif (LZ) is used for dimerization and the basic region (BR) is used for binding to specific DNA motifs (**Figure 1B**). This domain is highly conserved in the AP-1 family (3, 4), and the bZIP domain of Fra-1 is located at 107-161 amino acid sites. The Leucine zipper is composed of extended amino acids, with one leucine occurring every 6 amino acids. The dimer formed by the interaction between Fra-1 and JUN family, forms the “zipper” (**Figure 1C**) (26). The two subunits constituting the dimer form a continuous α helix, the carboxy-terminal region forms asymmetric helical coils, and the amino terminal region performs base-specific binding to DNA in the main groove (27). Because the amino acid composition in bZIP of FOS family members is slightly different from that of JUN family members, it cannot form homologous dimer with their own members like JUN family, but can only combine with JUN family members to form heterodimers and play a role (3). Moreover, Fra-1 can also heterodimers with other ubiquitous bZIP transcription factors (see **Table 1**) (13). In addition, related studies have found that since there is a wide electrostatic interaction network between subunits in the αhelix, Fos : Jun heterodimer has stronger affinity to DNA than Jun : Jun homodimer, and it is more stable and shows stronger transcriptional stimulation activity (38, 39). The dimer composed of Fra-1 and Jun preferentially binds to the DNA motif known as the TPA reaction element (TRE; also called AP-1 motif), it can also bind to cAMP-responsive element (CRE), although the latter has a slightly lower affinity than the former. The consensus sequences for TRE/AP-1 and CRE are 5′-TGA(C/G) TCA and 5′- TCACGTCA motifs, respectively (**Figure 1C**) (3, 26). In addition to the bZIP structure, FOS family members display a second highly homologous at the C-terminal, that is, the region where one of the two instability factors of resides. The C-terminal destabilizing domain (DEST) of Fra-1 protein containing 30-40 residues is necessary for its transformation activity and one of the reasons for its intracellular instability (**Figure 1B**) (40, 41).

**Table 1:** **|** Interaction protein of Fra-1 (HUMAN).

GENE name	Protein name	Reference
**JUNB**	Transcription factor JunB	(28–30)
**JUN**	Transcription factor Jun	(28, 31)
**ATF4**	Cyclic AMP-dependent transcription factor ATF-4	(32)
**CREB5-3**	Cyclic AMP-responsive element-binding protein 5	(29, 33)
**NDK7**	Nucleoside diphosphate kinase 7	(29, 33, 34)
**RNF11**	RING finger protein 11	(35)
**TAB2**	TGF-beta-activated kinase 1 and MAP3K7-binding protein 2	(29, 36)
**BATF3**	Basic leucine zipper transcriptional factor ATF-like 3	(29)
**KIFC3-5**	Kinesin-like protein KIFC3	(29)
**CCDC120**	Coiled-coil domain-containing protein 120	(29)
**GCC1**	GRIP and coiled-coil domain-containing protein 1	(29)
**NMDE3(GRIN2C)**	Glutamate receptor ionotropic, NMDA 2C	(35)
**PIN1**	Peptidyl-prolyl cis-trans isomerase NIMA-interacting 1	(29)
**Q0VDCB(FKBP1A)**	Peptidylprolyl isomerase	(35)
**HSP72(HSPA2)**	Heat shock-related 70 kDa protein 2	(35)
**CLAT-3(CHAT)**	Choline O-acetyltransferase	(35)
**FGFR3**	Fibroblast growth factor receptor 3	(35)
**USF1**	Upstream stimulatory factor 1	(29, 33)
**GELS(GSN)**	Gelsolin	(35)
**RASH(HRAS)**	GTPase HRas	(35)
**LDOC1**	Protein LDOC1	(30, 37)
**WFS1**	Wolframin	(35)
**KIF1B-2**	Kinesin-like protein KIF1B	(35)

The table was excerpted from Uniport (https://www.uniprot.org/uploadlists/).

Although these proteins belong to Fos family and share similar structure, the function and expression pattern actually not exactly the same. Skin sections show that Fra-1 is present in all layers other basal layer, and Fra-2 is detected in all layers with an increased level in the upper spinous layer. c-fos is present in the nuclei of the upper spinous and granular layer cells (42). This suggests that c-fos family proteins may exert different function in different types of cells in tissues. Moreover, Fra-1, but not Fra-2, orchestrates the inflammatory state of macrophages by regulating the expression of Arg1 and therefore impedes the resolution of inflammation (15). Another study demonstrated that Fra-1, Fra-2 and c-Fos promote the migration and invasion process of mammary carcinomas with different molecular target (43). In addition, it’s reported that c-Fos and Fra-1 have functional equivalence during vertebrate evolution, and Fra-1 rescues c-Fos-dependent functions such as bone development (44). However, c-Fos cannot totally replace Fra-1 because of the embryonic lethality in Fra-1-defecient mice. These findings show that the interaction and regulatory function among c-fos family proteins are very complicated and highly coordinated, and a detailed understanding of how these proteins determines its propensity to regulate cellular biological events remains further investigations.

Compared with other members of the FOS family, FOSL1 has very similar gene structures both in coding region and noncoding region, especially the size of the third exon and the protein encoded are completely the same (25). Although members of Fos family share similar structural features, there are also some differences. Firstly, in the mouse genome, individual genes of FOS family are not closely located. FOSL1 is on chromosome 19, c-Fos is on chromosome 12, and FosB is located on chromosome 7. In particular, the N-terminal and C-terminal of c-Fos and FosB have a transcriptional activation domain (TAD), while Fra-1 and Fra-2 are not found. At the beginning of discovery, due to the lack of transactivation domain, the entire Fra-1 protein fused with the DNA binding domain of Gal4 shows a lack of any transcriptional activation function (45), so it was once thought to be a transcription suppressor (39, 46, 47). But it was subsequently as a phosphorylation-dependent transcriptional activator (4). In many tumors, these non-transformed FOS proteins, especially Fra-1 and Fra-2, are involved in the progression of many tumors types (4). Thus, the structural characteristics of Fra-1 and the diversity and specificity of the combined dimers lay the foundation for its complex and diverse functions.

### 2.2 Fra-1 expression and regulation

Due to the important and diverse functions of FOS family members, precise and complex expression regulation is necessary to avoid the pathological effects of organisms. FOS and JUN are the main existing forms of AP-1 in mammals. The induced expression of ATF and MAF family proteins is more tissue/cell specific than FOS and JUN proteins (3). The expression level of Fra-1 was low in most tissues, but it was found to be quite high in testis and brain (25). In cells, AP-1 family members, including Fra-1, belong to immediate early cell genes (48). When fibroblasts are stimulated by serum, c-Fos and FosB are rapidly and transiently induced, Fra-1 and Fra-2 expressions are delayed but more stable, possibly through the activation of Fra-1 and Fra-2 promoters by Jun/Fos dimer (25, 49, 50). After stimulation, the expression of Fra-1 increased 12 times and lasted longer than that at rest (25). The results showed that Fra-1 was mainly localized in nucleus during indirect immunofluorescence assay (51). The expression characteristics of FOS family proteins may be the basis of their participation in the transition and asynchronous growth of G0-G1 cells (50)

#### 2.2.1 Transcriptional regulation

**Figure 2:**
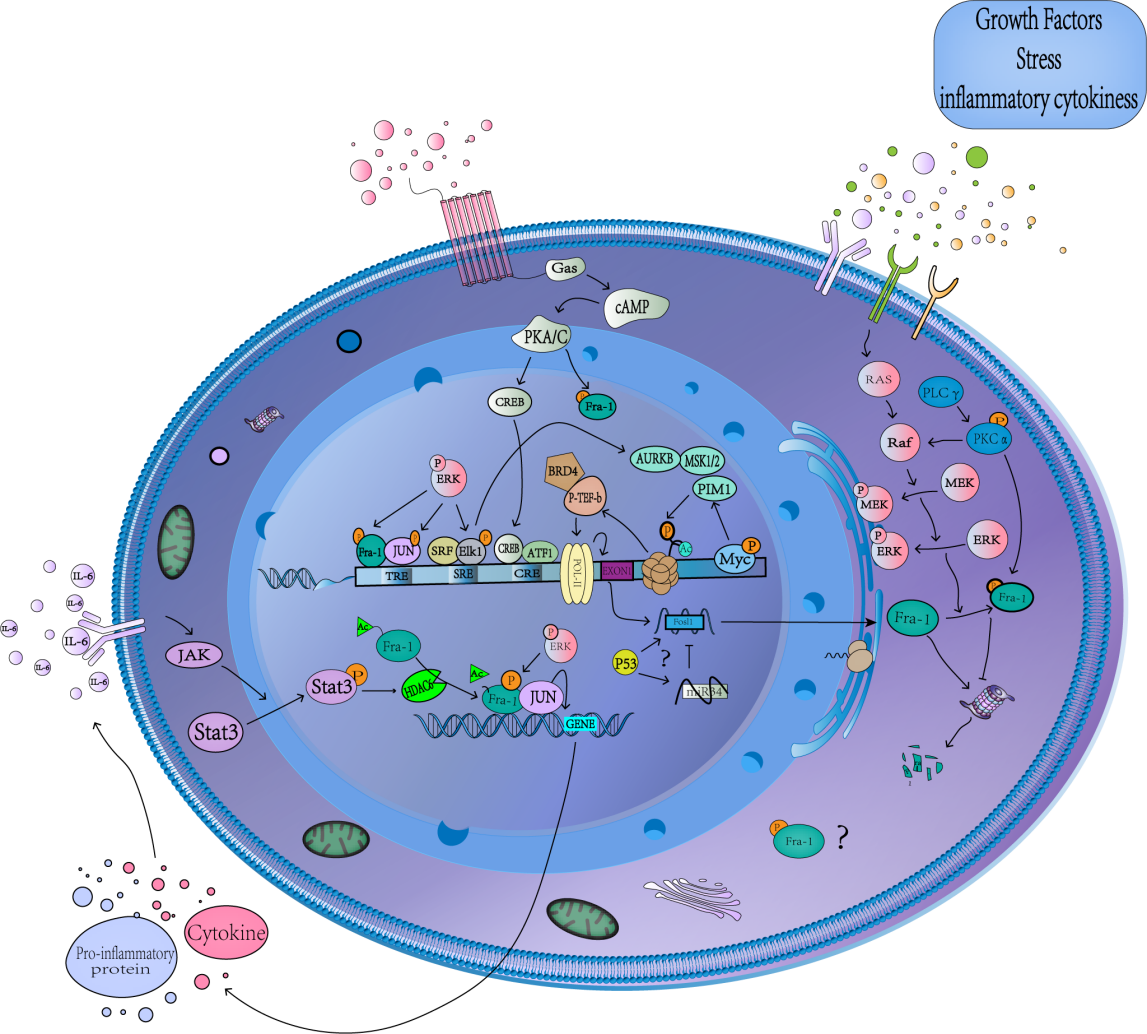
Transcriptional and posttranslational regulation of Fra-1. A schematic representation showing the multiple cellular pathways that converge to regulate Fra-1 function. Stimulation by various factors leads to MAPKs/cAMP/JAK-Stat3 activation, which in turn activates several TFs, causing the expression and phosphorylation of Fra-1, whose production regulation Fra-1 target gene expression. The unphosphorylated Fra-1 in the cytoplasm is easily degraded by proteasome. The question mark represents the unknow role of phosphorylated Fra-1 in the cytoplasm and the sepcific relationship between Fra-1, P53 and miR34. cAMP, Cyclic adenosine monophosphate; CRE, cAMP-responsive element; TRE, TPA reaction element; SRE, serum response element; PKA/C, protein kinase A/C; CREB, cAMP-response element binding protein; MSK1/2, Mitogen- and stress-activated kinases 1 and 2; ERK, extracellular regulated protein kinases; AURKB, Aurora Kinase B; P-TEFb, positive-elongation factor; BRD4, Bromodomain Containing 4; POL-II, polymerase II; ATF1, Activating Transcription Factor 1; Stat3, Signal transducers and activators of transcription 3; MAPK, mitogen-activated protein kinase; HDAC6, Histone Deacetylase 6; IL-6, interleukin-6; SRF, Serum response factor; JAK, janus kinase; RAS, rat sarcoma; Raf, Raf protein kinase; PLC, Phospholipase C.

The expression of Fra-1 involves the modification of several histones and recruitment of related proteins, and its transcriptional initiation is started after phosphorylation of S10 on H3 (**Figure 2**) (52). After receiving stimulation such as serum or growth factor, Myc binds to the corresponding DNA-binding domain and subsequently recruits Mitogen- and stress-activated kinases 1(MSK1/2) or proviral integration site for Moloney murine leukemia virus 1(PIM1) kinase to dephosphorylate H3S10 (52, 53), while TPA activates the extracellular regulated protein kinases(ERK) pathway to mediate Elk1 phosphorylation and TCF-SRE activation, Thus recruiting Aurora Kinase B (AURKB) to phosphorylate the site (54). Phosphorylation at H3S10 forces the 14-3-3 protein to be recruited to chromatin to recognize H3S10ph, which in turn recruits MOF to chromatin, leading to acetylation of H4K16 and H3K9. The P-TEFb/BRD4(positive-elongation factor/Bromodomain Containing 4) complex then recruits to the acetylation site, and catalyzes the phosphorylation of S2 in the carboxyl terminal domain of RNA pol-II(polymerase II), and finally leads to the release of stalled RNSD, resulting in transcriptional extension of FOSL1 (52, 54). In addition, Signal transducers and activators of transcription 3(Stat3) can be phosphorylated and acetylated in response to IL-6 stimulation and then bind to the promoter of FOSL1 to promote its transactivation (55). What’s more, FOSL1 has AP-1 site that allows it to be regulated by Fra-1 itself and other AP-1 family members (25, 56). Studies have found that tumor suppressor gene P53 can recruit to the first promoter region of FOSL1 to positively regulates its transcription, and FOSL1 is the only member of the FOS and JUN family with functional P53 binding sites (57–60).

#### 2.2.2 Post−translational regulation

Fra-1 is essentially an unstable protein, and the regulation of its stability may be the basis of its accumulation (61). Regulation of Fra-1 occurs at multiple levels, not only at the transcription and translation levels, but also under the influence of post-translational modification (such as phosphorylation, ubiquitination, etc.). Many proteins are known to be damaged through covalently linking ubiquitin chains to lysine residues at the n-terminal of the protein, which leads the protein to the proteasome, but there is a class of proteins that do not require prior ubiquitination to degrade (62–64). As mentioned above, Fra-1 is one of these non-ubiquitin-dependent proteasomal degrading proteins, which can be degraded even after all lysine residues are replaced by arginine or its N-terminal is blocked by fusion with Myc epitopes (51, 65).

Numerous studies have found that several kinases can phosphorylate Fra-1 *in vitro*, including cAMP-dependent kinase (PKA), protein kinase C (PKC), cyclin-dependent kinase 1-cdc2(cdc2) and mitogen-activated protein kinase (MAPK) (8, 41, 66–68). Fra-1 is the target of continuous activation of MAPK cascade, which can increase the transcription of FOSL1 (68), enhance the DNA binding activity of Fra-1 (66), and affect the transcription activity of Fra-1 after being activated (41). The activation of ERK has the greatest impact on Fra-1 content, and there is a linear relationship between the activity of ERK and the Fra-1 protein production rate in the same cell (60). ERK-based activity induces transcription of Fra-1, and its strongly increased activity is required for proteasome-dependent degradation of Fra-1 (20, 69, 70). When two serine residues S252 and S265 at C-terminal of Fra-1 protein are phosphorylated by ERK1/2, the DEST region at C-terminal of Fra-1 is inactivated, thus promoting the stability and accumulation of Fra-1, and the phosphorylation at both sites shows accumulation effect (51, 71). Therefore, under the stimulation of mitogen or in thyroid, colon and breast cancer cells, the high level of Fra-1 expression can be detected, where ERK pathway is highly active due to activation of upstream signal effector factors (51). Fra-1 can also be phosphorylated by protein kinase C (PKC) θ (S265, T223, T230, partially at T217 and T227 residues) and PKCα to prolong the half-time of Fra-1, its Synergistic phosphorylation with ERK-mediated phosphorylation stabilizes Fra-1 from degradation (28, 72, 73). The acetylation at the K116 site in the Fra-1 bZIP region inhibits the activity of Fra-1 and the binding ability to c-Jun independently of the protein stability mechanism. The Histone Deacetylase 6 (HDAC6)-mediated deacetylation at the Fra-1-K116 site can increase its DNA binding and transcriptional activity (74).

Studies have shown that downregulation of many tumor suppressor miRNAs leads to the accumulation of Fra-1 in cells. For example, miR34 can directly target Fra-1 to downregulate its expression, and P53 regulates Fra-1 expression through miR34 dependent manner (75, 76). However, some studies have found that P53 positively regulates the transcription of FOSL1 (57–60), and the specific mechanism remains to be explored. Several members of other tumor suppressor miRNA families (such as miR15/16) also target Fra-1 and affect the disease progression by regulating Fra-1 expression (see **Figure 2**) (77–79).

## 3 Fra-1-mediated immune regulation

The research of Fra-1 was focused on tumors and bone development. Generally, except for cervical cancer (80) and some controversial tumors (81–86), Fra-1 is highly expressed in most tumors and promotes malignant progression of them, control the proliferation and invasion of thyroid (87), bladder (88), lung and pancreatic cells (89). In mice, overexpression of Fra-1 leads to osteosclerosis, and conditional knockout leads to reduced bone mass (90). With the development of technology, there is increasing evidence that AP-1 controls the transcription of multiple inflammation-related genes, such as IL-6, IL-1β, and TNF-α, thereby regulating the inflammation immune response (15, 91, 92).

### 3.1 Macrophage

Macrophages are cellular components that exist in all tissues and bodies under homeostasis physiological conditions (93–95), which can change their phenotypes in response to many different stimuli. In the early 1990s, two distinct phenotypes of macrophage were described: one called classically activated (or inflammatory) macrophage (M1) and the other called vicariously activated (or wound-healing) macrophage (M2) (96, 97). Macrophages play a dual role in arthritis, myocardial infarction, or other inflammatory diseases, initiating and dispelling inflammation, and a mechanism is needed to program the macrophage phenotype (98). The role of the key transcription factor (TF) family in defining macrophage identity and controlling its function by inducing and maintain specific transcription processes has been fully established (99). Meanwhile, GO analysis of differentially expression genes between WT and Fra-1 deficient macrophage highlighted the important role of Fra-1 in macrophage (15).

**Figure 3:**
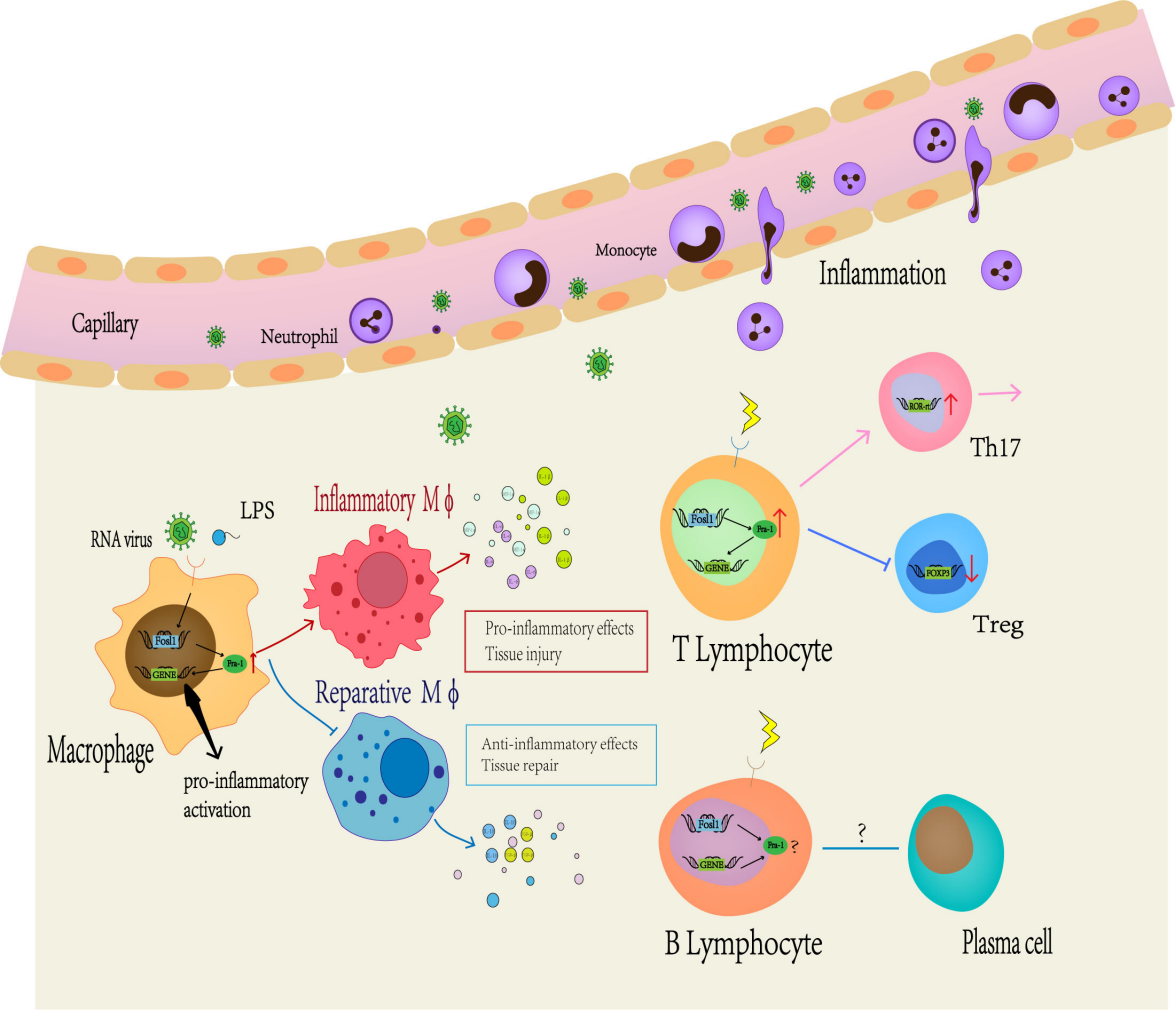
Fra-1 mediated immune regulation. A large number of studies have shown that Fra-1 regulates the function and activity of various immune cells *in vivo*. At first, the expression level of Fra-1 affects the migration of neutrophils, the differentiation of macrophages and T or B lymphocyte. When the body is infected or stimulated, the expression level of Fra-1 in immune cells increases, prompting more neutrophils to migrate from blood vessels to the site of inflammation, promoting M0 macrophages to differentiate more into M1-type macrophages and secrete more related pro-inflammatory cytokines. In addition, Fra-1 has been reported to be the core of T or B lymphocyte differention in the adaptive immune responses.

First, Fra-1 regulates the secretion of inflammatory cytokines in macrophages (**Figure 3**). In LPS-induced lung injury mice, Fra-1 is mainly expressed in alveolar macrophages (100), and LPS-induced transcriptional activation of Fra-1 is mediated by NF-kB and ERK1/2 signal transduction. Fra-1 selectively up-regulates LPS-induced NF-kB dependent pro-inflammatory cytokines (IL-1β, MIP-1A) and inhibits the anti-inflammatory response of alveolar macrophage (decreasing IL-10 expression) (20). Fra-1 can also affect the polarization state of macrophages. In myocardial infarction model, Leucine Rich Repeat Containing G Protein-Coupled538 Receptor 4 (Lgr4)-mediated cAMP/PKA/CREB pathway promotes transactivation of Fos in pro-inflammatory macrophage to enhance AP-1 activity, thereby triggering proinflammatory programming in macrophages to coordinate the proinflammatory state of infarcted macrophages (101). In the arthritis model, Fra-1 expression was up-regulated after LPS activated Toll-like receptors (TLR) cascade, and then it was directly bound to the promoter region of Arginase 1 (Arg1) to inhibit its transcription, making macrophages transform into M1 pro-inflammatory phenotype (15). Fra-1 also enhances osteoclast differentiation (102). In addition, it can also affect tumor progression by affecting macrophage activity: Breast cancer cells can overexpress Fra-1 in tumor-associated macrophages, induce macrophages to differentiate from M1 to M2, and then promote tumor immune escape, thereby promoting the invasiveness of breast cancer cells (21, 103). Signaling from CD137, a member of the tumor necrosis factor superfamily, can promote the expression of Fra-1, thus promoting the differentiation and migration of mononuclear macrophage into osteoclasts, then promoting the metastasis of tumor cells (104). Fra-1 also has a certain effect on the activation stage of macrophages. It has been demonstrated that Fra-1 can bind to Lipocalin 2 (Lcn2) promoter to promote neutropil gelatinase-associated lipocalin (NGAL) expression in immune tolerant BMDM(Bone marrow-derived macrophages), which has been described as a marker of inactivated macrophages (14).

### 3.2 Monocytes

Bone marrow precursors give rise to monocytes in blood (105). Together with tissue resident macrophage and dendritic cells (DCs), monocytes are classified as mononuclear macrophages (106), which can serve as a bridge between innate and adaptive immune responses. Some studies have shown that monocytes are highly plastic and can differentiate according to microenvironment changes (107, 108). Most articles use CD14, CCR2 and CD16 to classify monocyte subtypes, which are “classical” monocytes (CD14++CD16-CCR2++), “intermediate” monocytes (CD14++CD16+CCR2+) and “non-classical” monocytes (CD14+CD16++CCR2-) (109–111). Among them, the proportion of “classical” monocytes are the highest, which belongs to inflammatory cells that respond to many stimuli originated from damaged/infected tissue and produce inflammatory cytokines (111, 112); “intermediate” monocytes are highly phagocytic cells that produce high levels of ROS(reactive oxygen species) and inflammatory mediators (113, 114); and non-classical monocytes are reparative/patrolling cells that remove debris from vasculature and produce high levels of anti-inflammatory factors (115, 116). The researchers found that classical monocytes produced high levels of the broadest range of cytokines and chemokines in response to LPS, and this versability of classical monocytes is attributed to AP-1 transcription factors that are most highly expressed at the gene level by classical monocytes (117). Different combinations of the AP-1 transcription factor components are activated by different stimuli and signaling pathways, defining the transcription targets for AP-1 (118–120). What’s more, CD14+ monocytes can up-regulate the expression of Fra-1 to inhibit the production of type I proinflammatory cytokines monocytes and affect the effect of cells after stimulated by RNA virus (121).

### 3.3 Neutrophil

Neutrophils, which are a type of polymorphonuclear leukocyte, are well recognized as one of the major players during acute inflammation. They are typically the first leukocytes to be recruited to the inflammatory site and can eliminate pathogens through various mechanisms (122–125). Meanwhile, inappropriate recruitment can lead to tissue damage (126). Therefore, proper control of neutrophil activity and number is important for tissue homeostasis and progression. It has been reported that TGF-βsignal up-regulates the expression of Fra-1, c-Fos and c-Jun in neutrophil nucleus (127). In addition, in most inflammatory diseases, the overexpression of Fra-1 in mice will cause more neutrophils in blood vessels to migrate to tissue, aggravating tissue damage (128).

### 3.4 T lymphocytes

Traditionally, host immunity is divided into innate and adaptive immune responses. During infection, innate immunity is first stimulated (inflammation) and fully activated within minutes to hours (129). When innate immunity fails to eliminate pathogens, lymphocytes and adaptive immune mechanisms are activated, which can specifically recognize and eliminate pathogens, and it is important for host defense during late infection and secondary infection (130). Modulating adaptive immunity can play an important role in disease progression. T lymphocyte and B lymphocyte are the key cells of adaptive immune response, mainly responsible for the basic functions of cell-mediated immune response and antibody production respectively (131).

CD4+T lymphocytes can differentiate into different subtypes after being stimulated by cytokines, including Th1, Th2, Th9, Th17, Th22, T follicular helper (Tfh), and regulatory T cell (Treg). Under the regulation of intracellular transcription factors, they play different immune roles (132). Studies have shown that Fra-1 and other members of the AP-1 family play multifunctional roles in T cell development (7). In melanoma, Fra-1 is regulated by Ubc13-IKK signaling axis to maintain Treg cell function and inhibit its transformation into effector T cells (133). After overexpression of Fra-1 in CD4+T cells, it can act as a downstream target of Stat3 to induce the differentiation of Th17 cells (17). CD14+ monocytes can induce Fra-1 expression by activating MAPK through TLR7, and preferentially increase the production of Th17 polarizing cytokines to promote the polarization of Th17 cells (121). In the psoriatic model, Sobolev, V., et al. speculated that transcriptional regulation of PPARγ (Peroxisome Proliferator Activated Receptor Gamma) expression by the Fra-1 and Stat3/FOSL1 feedback pathways may be at the core of T cell differentiation (134).

IL-6 can promote the specific differentiation of naive CD4 + T cells, thereby linking congenital and acquired immune responses. The combination of IL-6 and transforming growth factor-β (TGF-β) is essential for the differentiation of Th17 cells (135). It can also inhibit TGF-β-induced Treg differentiation, and induces CD8 + T cells to differentiate into cytotoxic T cells (136). As mentioned above, Fra-1 plays an important role in the process of IL-6 secretion, and it is also the downstream effector molecule. Fra-1 may be the key transcription factor regulating T cell differentiation by these cytokines.

### 3.5 B lymphocytes

Blimp-1 is a transcription factor that plays an important role in the maturation of plasma cells from B cells to immunoglobulins and in the regulation of T cell homeostasis and tolerance (137). Studies have shown that c-Fos, a member of the Fos family, can positively regulate the expression of Blimp-1 and activate terminal differentiation of B cells (138), and its overexpression can also inhibit germinal center response (139). Increased AP-1 activity leads to early expression of Blimp-1, resulting in premature plasma cell formation and inhibition of germinal center B cell development (140, 141). However, subsequent studies have pointed out that Fra-1 can inhibit the production and development of plasma cells by regulating the direct combination with the Prdm1/Bimp1(PR/SET Domain 1/B lymphocyte induced maturation protein 1) promoter (142). In addition, the activation of B cells by TD antigen can reduce the expression of Fra-1, which can control the production of IgG-RF and inhibit the activation of osteoclasts driven by immune complexes (143). Therefore Fra-1 may be used as a regulator to block the negative effects of autoimmunity by inhibiting the formation of immune complexes. At the same time, in another study, stimulation of CD40 and surface Ig (sIg) receptors on B lymphocytes significantly induced Fra-1 expression by inducing protein kinase C (PKC) (144), which is not consistent with the previously observed phenotype. The regulation mechanism of Fra-1 expression in B cells still needs more research to clarify.

### 3.6 Cytokines

Cytokines are a kind of small molecule proteins with extensive biological activity synthesized and secreted by immune or some non-immune cells. They generally regulate cell growth, differentiation and effect by binding to corresponding receptors, and regulate immune response. Studies have found that Fra-1 plays an important role in the synthesis and secretion of various cytokines as well as the mechanism of action. Promoter regions of inflammatory cytokines and chemokines including TNF-α, IL-1β, IL-11, IL-8 and MCP-1 contain AP-1 binding sites (145, 146), but the reregulate mechanism of Fra-1 on cytokine expression is controversial.

We know that IL-6 mediates many inflammatory diseases and is considered to be an immune regulator that coordinates innate and adaptive immune responses (19). Morishita, H., et al. reported that overexpression of Fra-1 in RAW264.7 inhibited LPS-stimulated IL-6 production (147). In contrast, the study found that 4T1 breast cancer cells can promote the expression of Fra-1 in RAW264.7 in response to LPS and increase IL-6 production (21). Meanwhile, in LPS-triggered acute lung injury models, there was no significant difference in IL-6 production after knockout of Fra-1 in mouse alveolar macrophages (20). The effect of Fra-1 on cytokines is not absolute, which may be affected by cell types and the initial concentration of Fra-1 in cells, etc. The specific mechanism still needs to be further studied. Interestingly, Stat3 is a major transducer of signals triggered by cytokines and growth factors. However, Stat3 responds to the IL-6 signal on the membrane to phosphorylate downstream Fra-1, resulting in stable and increased intracellular Fra-1 content (55), which can further increase IL-6 expression. The expression of Fra-1 may be a positive feedback mechanism, which is similar to the phenotype that Fra-1 binds to its own promoter to promote expression. Furthermore, Du’s group reported that CCL-5, CCL-19 and CCL-21 could be regulated by Fra1 in medulla thymic epithelial cells (mTECs) and overexpression of Fra1 inhibited the transcriptional level of IL-1β, IL-6, IL-8 and ICAM1. They revealed that Fra1 disrupted inflammatory cytokine secretion by mTECs(medullary thymic epithelial cells) in the MG(myasthenia gravis) thymus (148). Another study conducted by Cho demonstrated that Fra-1-JunB complex directly binds to promoter region of *IL-17a* and activates its transcription (17). Ishihara et al. found that silencing Fra-1 by siRNA suppressed the expression of TNF-α and IL-1β in BV-2, a microglial cell line (17).

Taken together, these findings indicate that Fra-1 is intimately linked with the transcription of various cytokines in immune cells.

## 4 Immune-related diseases

The role of Fra-1 in the immune system enables it to regulate the occurrence and development of some diseases (see **Table 2**). It has been extensively documented in osteosclerosis and cancer-related diseases, which can be referred to other reviews (60, 71, 164, 165).

**Table 2:** Immune-related diseases.

Disease		Reference
**Sepsis**	–	(14, 149, 150)
**Cardiovascular system**	tumor angiogenesis	(151)
	vascular development	(152, 153)
	retinal disease	(154)
	atherosclerosis	(155)
	diabetic vascular restenosis	(156)
	myocardial infarction	(101)
**Respiratory system**	acute lung injury	(128)
	pulmonary fibrosis	(157, 158)
**Skin-related disease**	Psoriasis	(159, 160)
**Digestive system**	cholangitis	(161)
	liver fibrosis	(161)
	colitis	(162)
**Autoimmune disease**	acute graft-versus-host disease (aGVHD)	(163)
	autoimmune arthritis	(143)
	myasthenia gravis	(148).
**Tumor**	–	(21, 55, 74, 103, 104)

### 4.1 Sepsis

Sepsis is a life-threatening disease caused by high morbidity and mortality in hospitals (166). It is characterized by excessive inflammation in the early stage and subsequent abnormal regression of inflammation associated with long-term immunosuppression, which may eventually lead to multiple organ dysfunction. Proper control of inflammation plays an important role in the recovery of sepsis (167), which WHO has identified as a global health priority (168). Studies have proved that Fra-1 plays a certain role in the occurrence and development of sepsis. First, Matrix Metallopeptidase 9 (MMP9) was found to be one of the differentially expressed genes involved in the pathogenesis of sepsis (169). Down-regulation of MMP9 can reduce inflammation, while Fra-1 can occupy the promoter of MMP9 (170, 171). The silencing of Fra-1 destroys the expression of MMP9 (172), thereby reducing sepsis-induced renal inflammation and apoptosis (149). In addition, many studies have found that sepsis in mice overexpressing Fra-1 increases the severity of lung injury (150). In the immunosuppressive phase, specific knockout of Fra-1 in mouse macrophages leads to increased inflammation in immune tolerant mice, and Fra-1 can protect mice against endotoxin-induced sepsis by regulating NGAL (14). The regulation of Fra-1 on inflammation in sepsis is time-dependent, and the specific mechanism of action remains to be studied. Further studies are expected to find that Fra-1 can provide a new direction for sepsis treatment, and finally verify the clinical feasibility.

### 4.2 Cardiovascular system

Knockout of Fra-1 in mice results in embryonic death by blocking the angiogenesis of fetal placenta, indicating the importance of Fra-1 in angiogenesis. Prokineticin 2(PROK2)is an important contributor to tumor angiogenesis (151), Ring Finger Protein 213 (RNF213) is essential for normal vascular development (152), and Leucine Rich Alpha-2-Glycoprotein 1 (LRG1) plays a role in retinal vascularization abnormalities observed in oxygen-induced retinopathy (154). All three have been found to be regulated by Fra-1. It may also serve as a mediator of impaired pulmonary vascular development in neonates (153). In addition, Fra-1 plays a role in atherosclerosis (155), diabetic vascular restenosis (156), myocarditis, myocardial infarction (101) and other diseases. Previous studies suggest that the role of Fra-1 in regulating inflammatory angiogenesis in organs may be a topic for future research.

### 4.3 Respiratory system

Expression of Fra-1 is increased in the lungs of people stimulated by LPS or infected with bacteria *in vitro (* 100), and in the adult respiratory distress syndrome (ARDS) (173). Knockout Fra-1 in mice reduced LPS-induced acute lung injury and mortality (128), while overexpression showed opposite results (173). Studies have shown that Fra-1 plays a key role in inflammatory lung injury. Fra-1 plays a protective role in pulmonary fibrosis and regulates early fibro genic response. Fra-1 mediates anti-fibrosis effects by regulating pro-inflammatory, pro-fibrotic and fibrotic gene expression *in vivo* and *in vitro (* 157, 158). Conversely, Fra-2 has been studied as a causative factor for human fibrosis, and high expression of Fra-2 was observed in idiopathic and autoimmune-mediated pulmonary fibrosis samples (174). The structures of Fra-2 and Fra-1 are very similar, and the opposite effect of the two in the same disease is also worth thinking and studying, and both are considered to be therapeutic targets for pulmonary fibrosis.

### 4.4 Skin-related diseases

Psoriasis is a common inflammatory skin disease that is autoimmune and affects approximately 3% of the world’s population. It is characterized by keratinocyte proliferation, altered differentiation, and increased inflammation and angiogenesis (175). Fra-1 is very important for the stability of psoriasis inflammation, and is overexpressed human pathological tissues (159, 160). PPAR-γ inhibits the transcription of IL-17 by inhibiting its direct transcription factors RORC and STAT3, while FRA-1 can regulate the expression of STATA3, RORC and IL-17 by directly inhibiting PPARγ, thus regulating the development of psoriasis (134). Induction of Fra-1 overexpression in keratinocytes induces increased production of proinflammatory cytokines and chemokines (IL-8 and TNF-a), allowing subsequent immune cells (neutrophils and T cells) to migrate under this proinflammatory background and inducing plaque development in psoriasis (176).

### 4.5 Digestive system

The liver of Fra-1 overexpressed mice was progressively infiltrated by innate and adaptive immune cells. Compared with WT mice, the infiltrated cells in the liver of Fra-1 overexpressed mice were mainly composed of neutrophils and CD3+T cells, while B cells and macrophages rarely appeared in inflammatory sites, showing increased infiltration of activated CD4+CD69+ T cells. B cells, NK cells, and NKT cells decreased dramatically, while neutrophil infiltration increased (161). In DSS-induced colitis model, overexpression of Fra-1 inhibited the activation of NF-kB (162) and reduced the inflammatory response in mice.

### 4.6 Autoimmune disease

The absence of Fra-1 affects the inflammatory stage of arthritis, and the severity of the disease after inducing SIA in Fra-1 deficient mice is reduced. Different from the absence of other AP-1 members, c-Fos deficient mice have more severe arthritis (177). Overexpression of Fra-1 in CD4 + T cells can bind to JunB as a downstream target of Stat3 to induce Th17 cell differentiation and promote autoimmune arthritis (143). Inhibition of AP-1 activity can prevent acute graft-versus-host disease (aGVHD) by altering the differentiation of CD4 + T cells, such as reduced Th17/Th1 population and increased Treg population (163). Other studies have found that the expression of Fra-1 in medullary thymic epithelial cells (mTEC) of myasthenia gravis (MG) patients is increased, and its overexpression may destroy the secretion of inflammatory cytokines in mTEC of MG patients’ thymus. accompanied the increase of CCL5, CCL-19 and CCL-21, and the decrease of ICAM1, IL-6, IL-1β (148).

### 4.7 Tumor

Numerous studies have confirmed that Fra-1 is crucially involved in human tumor progression and metastasis, thus representing a promising therapeutic target (178, 179). As we all know, inflammation and cancer is intimately linked, the tumor microenvironment (TME) plays a prominent role in the growth of tumor cells. As the major inflammation component of the TME, M2d macrophages are educated by the TME such that they adopt an immunosuppressive role that promotes tumor metastasis and progression (180). Fra-1 can increase the production of the cytokine IL-6 and skew RAW264.7 macrophage cell differentiation into M2d macrophage, then promotes tumor metastasis and progression (21, 103); CD137 promotes the migration of monocytes/macrophages to tumor microenvironment by upregulating the expression of Fra1. It also promoted the differentiation of monocytes/macrophages into osteoclasts at the same time, thus providing a favorable microenvironment for the colonization and growth of breast cancer cells in bone (104); The cytokine IL-6 induces Fra-1 deacetylation, then promoting colorectal cancer stem-like properties (74); In addition, the existence of an aberrant IL-6/STAT3/Fra-1 signaling axis leading to colorectal cancer(CRC) aggressiveness through EMT induction, which suggests novel therapeutic opportunities for the malignant disease (55). A DNA vaccine encoding transcription factor Fra-1 and secretory IL-18 induces a long-lived memory T-cell response which can contribute to tumor regression (181–183). The effect of Fra-1 on immune cells also plays an important role in tumorigenesis.

## 5 Fra-1 related treatment

The complex and diverse functions of AP-1 have made it an actively pursued drug target in past studies, but transcription factors have been considered difficult to target because their activity is triggered by protein-protein or protein-DNA interactions. However, inhibiting key transcription factors such as c-Myc (184, 185), which is previously considered to be drug-free, and then confirmed, this provides confidence and foundation for the suppression of other transcription factors such as AP-1. Selective AP-1 inhibitor T5224 (61) (3-propionic acid (T-5224)) has been investigated in phase II clinical trials for novel c-Fos/AP-1 inhibitors for rheumatoid arthritis (186). Some studies have found that some tumor prevention drugs, such as resveratrol (187), green tea (188) and curcumin (189), work by inhibiting Fra-1 expression in the model system. Retinol like SR11302 inhibits AP-1 activity and FOSL1 expression without activating RARE transcription (12, 190–194). SR11302 inhibits alloreactive T cell response in a dose-dependent manner, showing decreased Th17/Th1 population and increased Treg population *in vivo (* 163). Meanwhile, FOSL1 was found to be the target of anti-epileptic drug levetiracetam (LEV) for inhibiting neuroinflammation. LEV can reduce the expression of FOSL1 in microglia to inhibit its activation and thus inhibit the inflammatory response involved (195). At present, there is no specific small-molecule inhibitor of Fra-1 that is effective both in cells and animals. In fact, the existing selective inhibitors are not only act on Fra-1, which would elicit undesired side effects. As extensive screening of specific inhibitors of Fra-1 is still ongoing, additional improvements may be achieved from future experimentation. Another strategy for targeting Fra-1 is genetic inhibition. Vicent’s group reported that deficiency for Fra-1 in a genetically engineered mouse model of Cholangiocarcinoma using constitutive and inducible short-hairpin RNAs extended mouse survival by decreasing the oncogenic potential of transformed cholangiocytes (179).

Sometimes directly targeting Fra-1 may be difficult at present, but if more targets for Fra-1 regulation can be found, it may be relatively easy to control the target gene and obtain the corresponding phenotype. For example, the expression of the Mevalonate pathway gene HMGCS1 is controlled by direct FOSL1 promoter binding. Genetic and pharmacological inhibition of HMGCS1 and AURKA leads to loss of FOSL1, which can reduce the carcinogenic potential of transformed bile duct cells and prolong mouse survival (179). As previously mentioned, MAP kinase pathway plays a role in the content and stability of Fra-1 in cells. Inhibition of ERK1/2 pathway can block the recruitment of c-Jun and NF-kB transcription factors to endogenous FOSL1 promoter after LPS stimulation to reduce Fra-1 mRNA expression, thereby reducing the expression of inflammatory factors (20). The progression of many diseases is influenced by the immune system, and the role of Fra-1 in the immune system makes it a potential target for disease treatment. The regulation of Fra-1 and its pre- and post-effectors has a great impact on the outcome of the disease. However, the mechanism of Fra-1 in immune cells and immune responses is still unclear, which needs more research to elaborate, and then it is expected to develop effective drugs for clinical use.

## 6 Discussion

As a member of FOS family, Fra-1 plays an important role in cell proliferation, differentiation, tumor transformation, immunity, inflammation and other processes. AP-1 is an important nuclear transcription factor. FOS and JUN families, which mainly exist in mammals, bind to form heterodimers through the leucine zipper domain to regulate the transcription of related target genes. It is involved in MAPK (196), Wnt (197) and other important signaling pathways, which accumulate in cells mainly through transcription and post-translational modification. Since tumor progression is closely related to the intensity of immune response, when most studies focus on the role of Fra-1 in tumors, the other part focuses on the function of Fra-1 in immunity.

Fra-1 affects the differentiation and activation of immune cells, especially macrophages, as well as the secretion of many cytokines (17, 121, 133, 138, 142, 198, 199), but the specific mechanism remains to be further explored. The regulation of the immune response of Fra-1 makes its intracellular expression affect the outcome of many diseases, such as sepsis (149), atherosclerosis (155), myocardial infarction (101), psoriasis (134), colitis (162), etc. This evidence points at the importance of Fra-1 in immune regulation. Thus, it’s necessary to figure out the molecule controls of Fra-1 that maintain the steady-state homeostasis of immune cells and how dysregulation of these processes could precipitate a wide range of immune-associated disorders. Meanwhile, targeting Fra-1 is expected to be an effective intervention strategy. However, there are still many basic questions about Fra-1 that cannot be answered.

First, why do the same structural molecules play the opposite effect. The structures of most FOS family members are similar, especially Fra-1 and Fra-2, both of which lack the trans-activation domain at their C-terminus (45), but sometimes they show different results in the same disease. For example, Fra-1 has a protective effect on pulmonary fibrosis, but Fra-2 is the pathogenic factor of the disease (157, 158, 174). Secondly, whether the Fra-1/AP-1 transcription factor only binds to the promoter of the target gene plays a role. Prior to the emergence of ‘omics’ research, the molecular transcription research of AP-1 was basically concentrated in the gene promoter region. This view has recently been challenged by genome-wide studies showing that AP-1 frequently binds to enhancer sites far from the gene transcription start site (TSSs), although the nature of this has not been determined (13, 31). It has been found that FOSL1 not only acts on promoters of target genes, but also selectively binds to its enhancers and corresponding Mediators to establish super enhancers (SEs), thus promoting the expression of target genes (12). At present, our understanding of Fra-1 targeted gene and its transcriptional regulation mechanism is still limited. It is not clear whether Fra-1 affects chromatin or whether it affects the expression of target genes in cells by using the preset 3D structural network. Then, whether Fra-1 must be incorporated into the nucleus through transcription factors with other proteins to function. The activation of ERK by Nitrogen mustard (NM) increases Fra-1 levels in mouse epidermis and cultured human keratinocytes, where Fra-1 is mainly expressed in the cytoplasm rather than the nucleus, which contributes to IL-8 expression in NM-damaged skin (23).

In summary, a large number of studies have found that Fra-1 plays a certain role in the immune response process. Intervention at the levels of transcription, translation and post-translational modification can regulate its expression in cells, thereby regulating some cell activities and molecular production. However, the specific mechanism remains unclear, and many problems still need further study.

## Author contributions

Y-YH, H-FZ, and LC contributed to the conceptual design, writing, and editing for this manuscript. Y-TW, W-LX, Z-ZX, YX, Y-QF, G-YL, XL, and JL provided valuable discussions and comments. Q-PW revised the manuscript and commented on previous versions of the manuscript. All authors contributed to the article and approved the submitted version.

## Funding

This work was supported by the National Natural Science Foundation of China (Grant No. 81901948), the National Natural Science Foundation of China (Grant No. 81873952), and the National Key Research and Development Program of China (Grant No. 2018YFC2001900).

## Conflict of interest

The authors declare that the research was conducted in the absence of any commercial or financial relationships that could be construed as a potential conflict of interest.

## Publisher’s note

All claims expressed in this article are solely those of the authors and do not necessarily represent those of their affiliated organizations, or those of the publisher, the editors and the reviewers. Any product that may be evaluated in this article, or claim that may be made by its manufacturer, is not guaranteed or endorsed by the publisher.

## Glossary

**Table:**

AP-1	activating protein-1
Fra-1	Fos-related antigen 1
FOSLl	FOS Like 1
bZIP	basic Leucine zipper
BR	basic region
TRE	TPA reaction element
cAMP	Cyclic adenosine monophosphate
CRE	cAMP-responsive element
SRE	serum response element
DEST	C-terminal destabilizing domain
TAD	Transcriptional activation domain
MSK1/2	Mitogen- and stress-activated kinases 1 and 2
PIM1	(proviral integration site for Moloney murine leukemia virus 1)
SRF	Serum response factor
ERK	extracellular regulated protein kinases
RAS	rat sarcoma
Raf	Raf protein kinase
AURKB	Aurora Kinase B
P-TEFb	positive-elongation factor
BRD4	Bromodomain Containing 4
POL-II	polymerase II
ATF1	Activating Transcription Factor 1
Stat3	Signal transducers and activators of transcription 3
PKA	protein kinase A
PKC	protein kinase C
cdc2	cyclin-dependent kinase 1-cdc2
MAPK	mitogen-activated protein kinase
HDAC6	Histone Deacetylase 6
TF	transcription factor
MMP	matrix metalloproteinase
IL-6	interleukin-6
MIP-1A	Macrophage inflammatory protein 1α
Lgr4	Leucine Rich Repeat Containing G Protein-Coupled Receptor 4
CREB	cAMP-response element binding protein
TLR	Toll-like receptors
Arg1	Arginase 1
Lcn2	Lipocalin 2
NGAL	neutropil gelatinase-associated lipocalin
BMDM	Bone marrow-derived macrophages
PPARγ	Peroxisome Proliferator Activated Receptor Gamma
Blimp-1	B lymphocyte induced maturation protein 1
Prdm1	PR/SET Domain 1
PROK2	Prokineticin 2
RNF213	Ring Finger Protein 213
LRG1	Leucine Rich Alpha-2-Glycoprotein 1
aGVHD	acute graft-versus-host disease
mTEC	medullary thymic epithelial cells
MG	myasthenia gravis
TSSs	transcription start site
SEs	super enhancers
JAK	janus kinase
PLC	Phospholipase C.
